# A rare case of giant mediastinal thymolipoma in an 18‐year man

**DOI:** 10.1002/ccr3.8530

**Published:** 2024-02-19

**Authors:** Hourieh Soleimani, Behzad Aminzadeh, Ehsan Hassannejad, Asma Payandeh, Batul Oudi, Neda Karimabadi

**Affiliations:** ^1^ Department of Radiology, Faculty of medicine Mashhad University of Medical Sciences Mashhad Iran; ^2^ Department of Radiology, School of Medicine Birjand University of Medical Sciences Birjand Iran; ^3^ Faculty of Medicine Mashhad University of Medical Sciences Mashhad Iran; ^4^ Pathology Department, Faculty of Medicine Mashhad University of Medical Sciences Mashhad Iran

**Keywords:** lipoma, mediastinum, neoplasm, radiology, thymolipoma

## Abstract

Thymolipoma is a rare benign thymic lesion that can manifest as a sizable anterior mediastinal mass. Considering their rarity and challenging preoperative diagnosis, it is crucial to consider these tumors when dealing with anterior mediastinal masses.

## 1 INTRODUCTION

Thymolipoma is a rare pathological entity that grows slowly and is benign in nature, predominantly found in the anterior mediastinum. It is comprised of mature adipose cells and thymic tissue. Thymolipoma represents a proportion of 2%–9% among all thymic neoplasm.^1^

With a slow growth pattern, these tumors are commonly identified when they generate symptoms of pressure or are incidentally detected during the assessment of other complaints.^2^ Conditions such as chronic lymphocytic leukemia, myasthenia gravis, aplastic anemia, hyperthyroidism, and Hodgkin's disease have been found to be associated with this tumor. Complete surgical excision remains the preferred choice for treatment.^1^ We hereby present a case of a patient with a substantial mediastinal mass that was confirmed to be a thymolipoma. The case was introduced based on the exceptional rarity and monumental size of a mediastinal mass.

## 2 CASE PRESENTATION

### 2.1 Case history and examination

An 18‐year‐old male, who had a previous hospitalization at the age of five due to meningitis and no other medical history, was admitted to the hospital after being involved in a car accident. The patient had no history of positive family background or drug use. The patient's vital signs remained stable, and there were no complaints of dyspnea. Oxygen saturation was 98%. The patient mentioned chest pain that occurred after the trauma.

The physical examination revealed limited chest movement, decreased tactile vocal fremitus on the right side, and dullness upon percussion over the right area. A decrease in breath sounds on the right side was detected during chest auscultation.

### 2.2 METHODS

**FIGURE 1:**
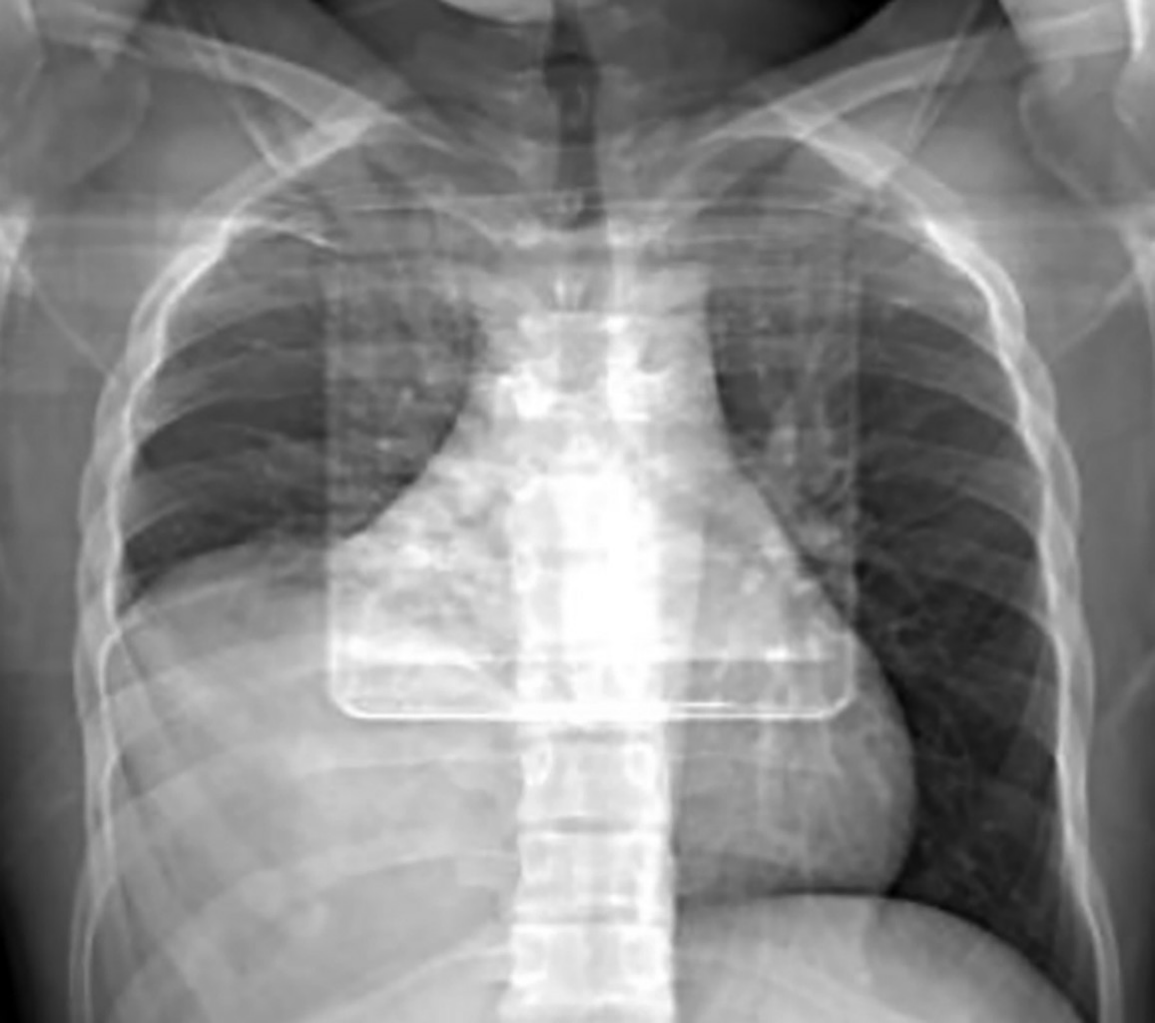
Chest CT scout view shows an opacity in the right lower hemithorax with minimal shifting of the heart towards the left side.

A chest CT scan was performed on the patient. The CT scout view findings demonstrated the presence of an opacity in the right lower hemithorax without clearly evident shifting of the heart towards the left side (Figure 1).

**FIGURE 2:**
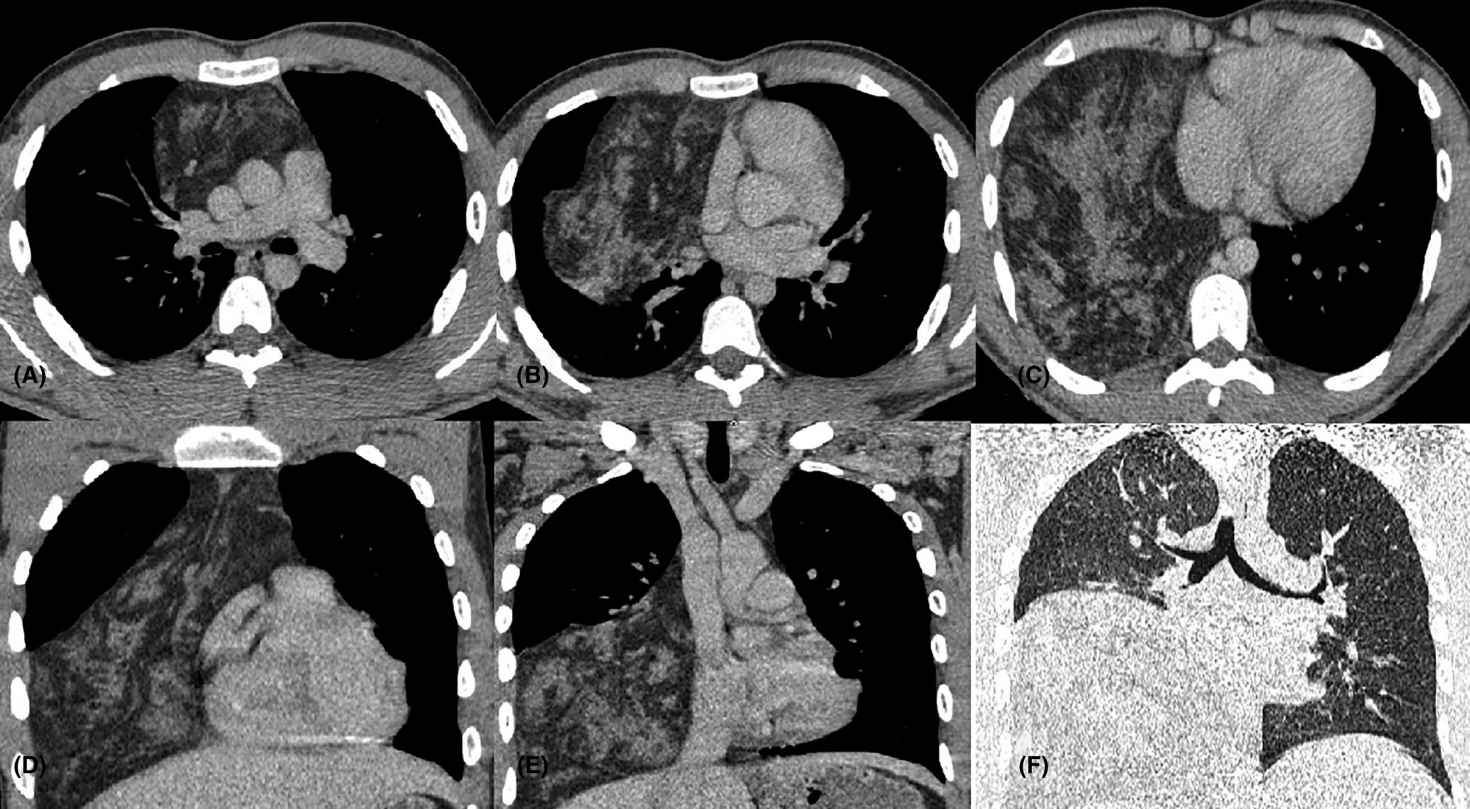
Chest CT scan shows a predominantly fat‐containing mass with soft tissue components in axial (A–C) and the coronal plane (D–E), appearing to originate from the anterior mediastinum and extending into the right hemithorax, causing a shift of the heart and mediastinum to the left. (F) shows near complete collapse of lower lobe of right lung.

The CT scan revealed the presence of a predominantly fat‐containing mass with soft tissue components, measuring approximately 13 × 23 × 15 cm. This mass appeared to originate from the anterior mediastinum and extended into the right hemithorax, without a clearly evident shift of the heart and mediastinum to the left. The lesion did not reach below the diaphragm. The lower lobe of right lung exhibited almost complete collapse (Figure 2).

**FIGURE 3:**
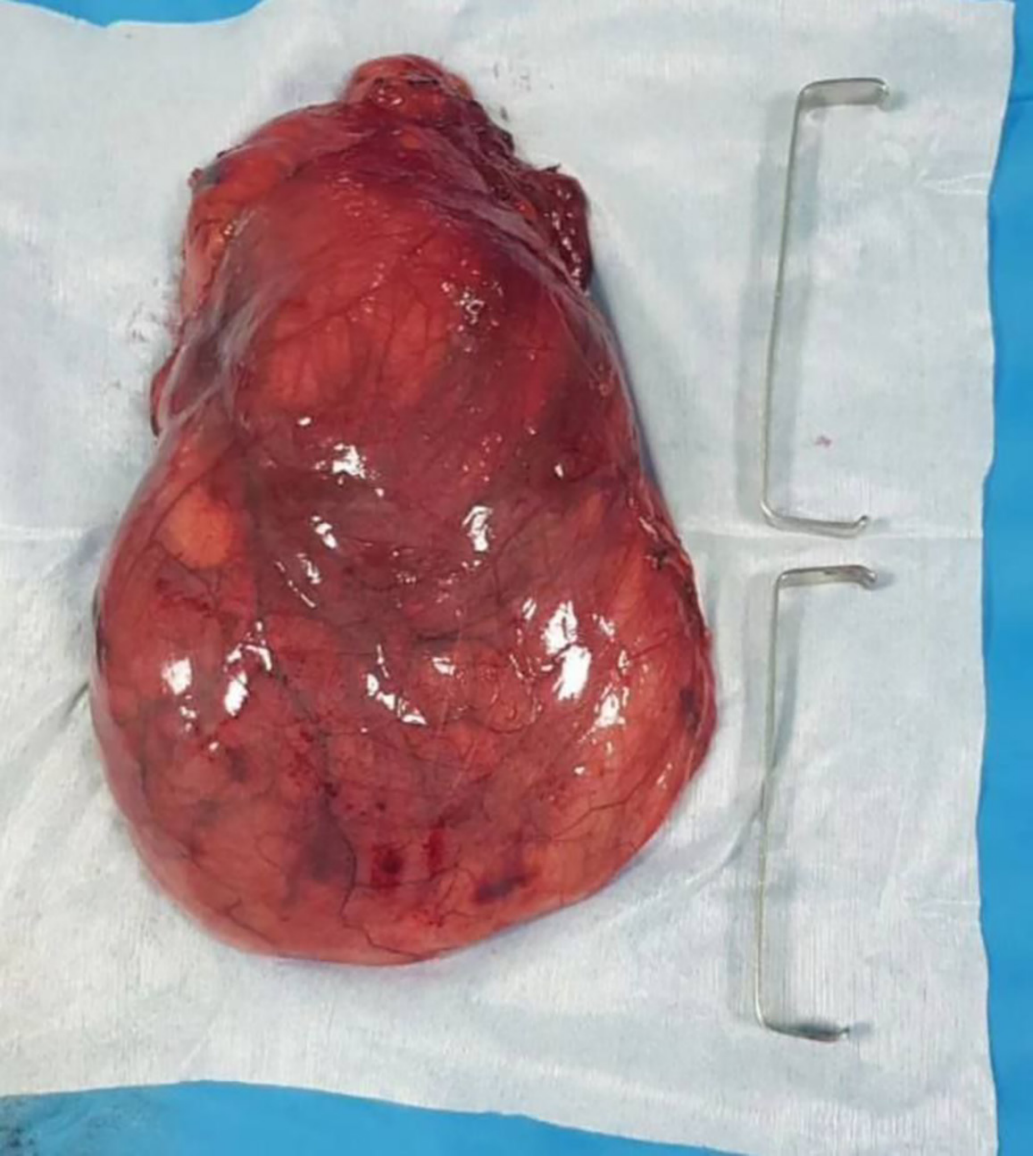
Intraoperative view of excised large mass shows multilobulated appearance.

The patient was planned for surgery. Through a right thoracotomy, the mass was entirely excised (Figure 3).

**FIGURE 4:**
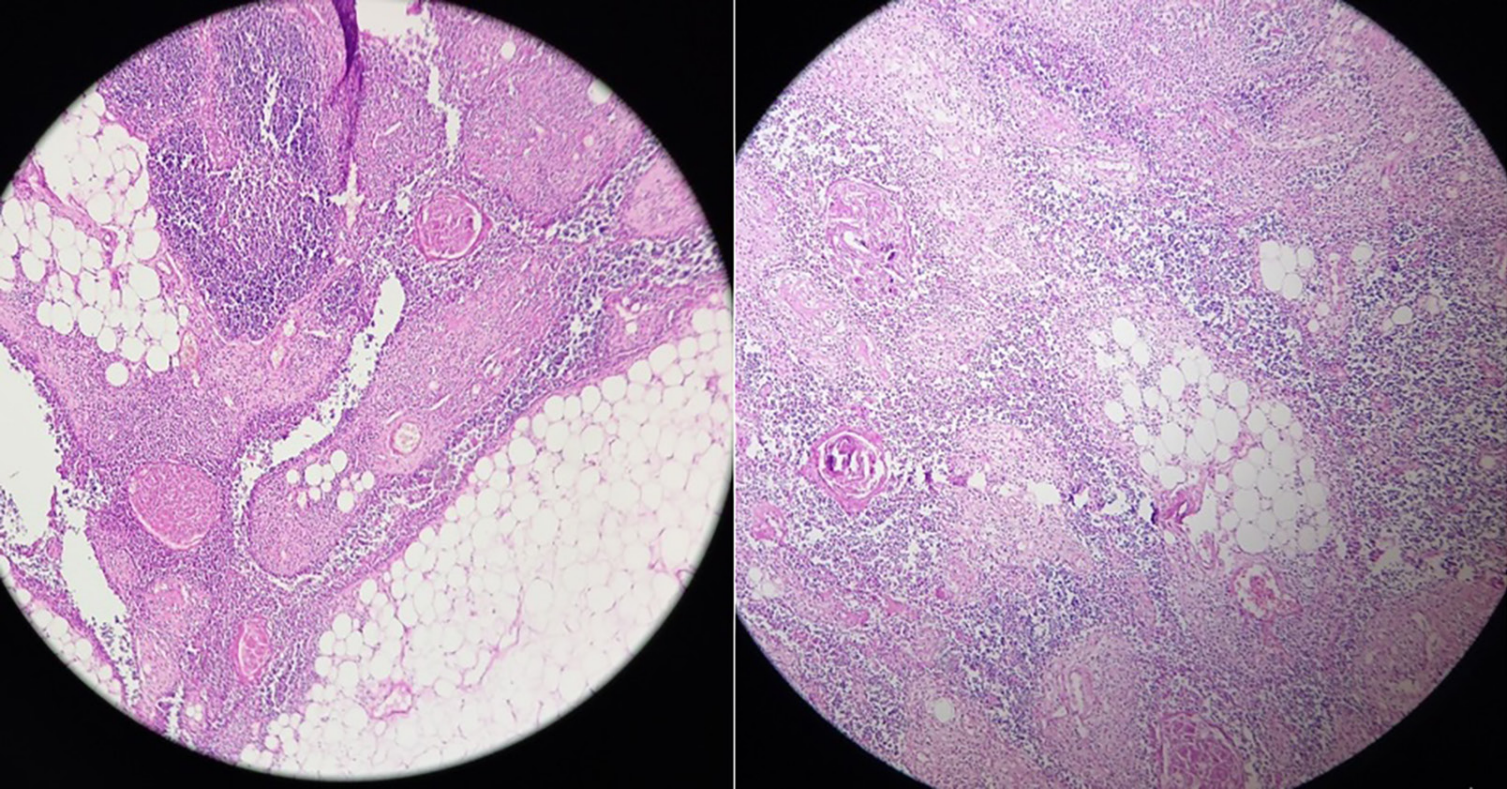
Microscopic appearance shows thin fibrous capsule surrounding lobules of mature non‐atypical adipose tissue with islands of non‐neoplastic thymic tissue with Hassall's corpuscle.

The histopathological examination revealed the presence of an encapsulated lesion comprising mature adipose tissue that contained islands of non‐neoplastic thymic epithelial cells (Figure 4). The final diagnosis was thymolipoma.

### 2.3 CONCLUSION AND RESULTS

The patient experienced no postoperative complications and was discharged in excellent condition on the 11th day following the surgery.

## 3 DISCUSSION

Thymolipomas, a type of mediastinal tumor that consists of mature adipose and thymic tissue, are exceedingly rare and arise in the thymus gland. They make up 1.1% of all solid mediastinal tumors and do not exhibit any gender preferences.^3^ Thymolipomas are characterized by the presence of abundant mature fat, which separates the thymic tissue component, with no evidence of atypia or mitotic activity. Although most of these tumors are clinically quiescent, they can grow to significant sizes and present clinical symptoms such as compression of the lower respiratory tree, resulting in breathlessness, coughing, chest discomfort, and upper respiratory tract infection.^2^ Furthermore, it can lead to cardiac compression and subsequently, chronic heart failure.^4^ The radiographic characteristics have the ability to resemble various conditions, such as cardiomegaly, pericardial effusion, pleural tumors, pericardial tumors, and basal atelectasis.^5^, ^6^

Thymolipoma has been found to have associations with chronic lymphocytic leukemia, myasthenia gravis, aplastic anemia, hyperthyroidism, and Hodgkin's disease in certain cases.^7^ In our scenario, there was no relationship established between this presenting case and the tumors mentioned earlier.

The CT scan is typically the preferred diagnostic modality. The consideration of thymolipoma diagnosis should be taken into account when evaluating the accuracy of an anterior mediastinal mass characterized by fatty tissue containing soft tissue streaks, which signify islands of normal thymic components, along with contralateral displacement of the mediastinum on CT scans.^8^, ^9^ A similar finding was observed in the CT scan outlined in this case report. No evident contralateral displacement of the mediastinum was seen in our case that may be due to the characteristic soft nature of this tumor; however it is imperative to investigate additional studies to shed light on this issue. Teratoma, lipoma, lipomatosis, and liposarcoma are potential differential diagnoses that should be considered.^3^ The characteristic appearance of teratoma on CT scan is that of a well‐defined cystic lesion with the presence of fluid, soft tissue, and fat attenuation. Calcifications also may be observed and a tooth or a fragment of bone is rarely seen.^10^ The CT scan findings of a lipoma include homogeneous fat attenuation, absence of contrast enhancement, and clearly defined margins. CT shows lipomatosis as uniform and extensive fat‐attenuation material surrounding anatomical structures without causing invasion or compression. CT imaging reveals a mediastinal liposarcoma as a mass that displays heterogeneous enhancement and contains varying amounts of fat and soft‐tissue density.^11^

There have been reported cases of thymolipoma in different age groups.^1^, ^2^, ^6^, ^8^, ^9^, ^12^ Thymolipomas, although uncommon, should be included in the differential diagnosis, even in cases of infants with an anterior mediastinal mass. A case of this type of tumor occurring in a 6‐month‐old boy has been reported.^13^ Thymic tumors, specifically thymoma, are rarely found outside the mediastinum,^14^, ^15^ but the occurrence of thymolipoma in the lung or other mediastinal structures, excluding the thymus, has not been recorded. The considerable dimensions of the tumor in our patient presented considerable obstacles in determining the precise location or origin of the mass before the operation.

Surgical excision is the recommended treatment for thymolipoma, as it is curative and eliminates the need for long‐term follow‐up for a benign tumor. No cases of recurrence, metastasis, or mortality have been reported.

To summarize, thymolipoma is a rare noncancerous thymic abnormality that may manifest as a sizable mass within the mediastinum and is typically detected during the evaluation of a secondary disease. The prognosis for this tumor is excellent after surgical excision due to its benign nature. Given their rarity and challenging preoperative diagnosis, it is important to always consider these tumors when managing anterior mediastinal masses.

## AUTHOR CONTRIBUTIONS

**Hourieh Soleimani:** Conceptualization; data curation; investigation; project administration; supervision; writing – original draft; writing – review and editing. **Behzad Aminzadeh:** Data curation; supervision; writing – original draft. **ehsan hassannejad:** Data curation; validation; writing – original draft; writing – review and editing. **Asma Payandeh:** Data curation; writing – original draft; writing – review and editing. **Batul Oudi:** Data curation; writing – original draft. **Neda Karimabadi:** Conceptualization; data curation; methodology; project administration; writing – original draft.

## FUNDING INFORMATION

No fund was available for this study.

## CONFLICT OF INTEREST STATEMENT

The authors declare no conflict of interest in this study.

## ETHICS STATEMENT

The patient has provided written informed consent for the publication of this case report.

## CONSENT

Written informed consent was obtained from the patient for publication of this case report and any accompanying images.

## Data Availability

The data that support the findings of this study are available on reasonable request from the corresponding author. The data are not publicly available due to privacy or ethical restrictions.
